# A patient with two gliomas with independent oligodendroglioma and glioblastoma biology proved by DNA-methylation profiling: a case report and review of the literature

**DOI:** 10.1007/s10014-021-00423-0

**Published:** 2022-01-11

**Authors:** Theo F. J. Kraus, Christoph Schwartz, Lukas Machegger, Barbara Zellinger, Dorothee Hölzl, Hans U. Schlicker, Johannes Pöppe, Barbara Ladisich, Mathias Spendel, Michael Kral, Karl Sotlar

**Affiliations:** 1 Institute of Pathology, University Hospital Salzburg, Paracelsus Medical University, Müllner Hauptstr. 48, 5020 Salzburg, Austria; 2 Department of Neurosurgery, University Hospital Salzburg, Paracelsus Medical University, Ignaz-Harrer-Str. 79, 5020 Salzburg, Austria; 3 Institute of Neuroradiology, University Hospital Salzburg, Paracelsus Medical University, Ignaz-Harrer-Str. 79, 5020 Salzburg, Austria

**Keywords:** Biomarker, Glioma, Oligodendroglioma, Glioblastoma, DNA-methylation profiling

## Abstract

Here, we report on a patient presenting with two histopathologically distinct gliomas. At the age of 42, the patient underwent initial resection of a right temporal oligodendroglioma IDH mutated 1p/19q co-deleted WHO Grade II followed by adjuvant radiochemotherapy with temozolomide. 15 months after initial diagnosis, the patient showed right hemispheric tumor progression and an additional new left frontal contrast enhancement in the subsequent imaging. A re-resection of the right-sided tumor and resection of the left frontal tumor were conducted. Neuropathological work-up showed recurrence of the right-sided oligodendroglioma with features of an anaplastic oligodendroglioma WHO Grade III, but a glioblastoma WHO grade IV for the left frontal lesion. In depth molecular profiling revealed two independent brain tumors with distinct molecular profiles of anaplastic oligodendroglioma IDH mutated 1p/19q co-deleted WHO Grade III and glioblastoma IDH wildtype WHO grade IV. This unique and rare case of a patient with two independent brain tumors revealed by in-depth molecular work-up and epigenomic profiling emphasizes the importance of integrated work-up of brain tumors including methylome profiling for advanced patient care.

## Introduction

The 2016 World Health Organization (WHO) classification of tumors of the central nervous system (CNS) integrates, both, histology and molecular pathology as integrated aspects of brain tumor classification [8]. Thereby, DNA-methylation analysis is a promising novel technology for accurate brain tumor classification since previous studies revealed that distinct methylation profiles define distinct brain tumor entities with high accuracy [2, 3, 5, 9, 10, 12]. One of the most prominent examples is the inclusion of *isocitrate dehydrogenase* (*IDH*) *1* and *2* status, and loss of chromosomes 1p and 19q as integrated parts of the classification of glioma: Since 2016 the diagnosis of astrocytomas requires the analysis of *IDH* mutation status, and the diagnosis of oligodendrogliomas requires the assessment of both *IDH* mutations, as well as combined 1p/19q losses. [8] Thereby, oligodendrogliomas *IDH* mutated 1p/19q co-deleted show significantly better overall survival compared to astrocytomas *IDH* mutated and glioblastomas *IDH* wildtype [8].

Gliomas show a typical diffusely infiltrating growth pattern into surrounding brain tissue and recurrences after initial resection/treatment. Importantly, it has been established that the molecular features of gliomas, i.e. *IDH*-, 1p/19q- and *TERT*-Status, do not change during tumor recurrence and/or progression [8]. The distinct molecular background of astrocytomas WHO grade II and III as well as secondary glioblastomas WHO grade IV can be proven by revealing *IDH1* mutations in codon 132 and *IDH2* mutations in codon 172 [8]. The molecular background of oligodendrogliomas WHO grades II and III can be confirmed by demonstrating combined *IDH1/2* mutations and chromosomal losses on 1p and 19q [8]. In contrast to the aforementioned gliomas, primary glioblastomas show *IDH1/2* wildtype status [8].

Here, we report on a 42 years old patient with two brain tumors that showed distinct molecular patterns in integrated work-up and epigenomic profiling proving independent tumor origins.

## Clinical summary

**Fig. 1:**
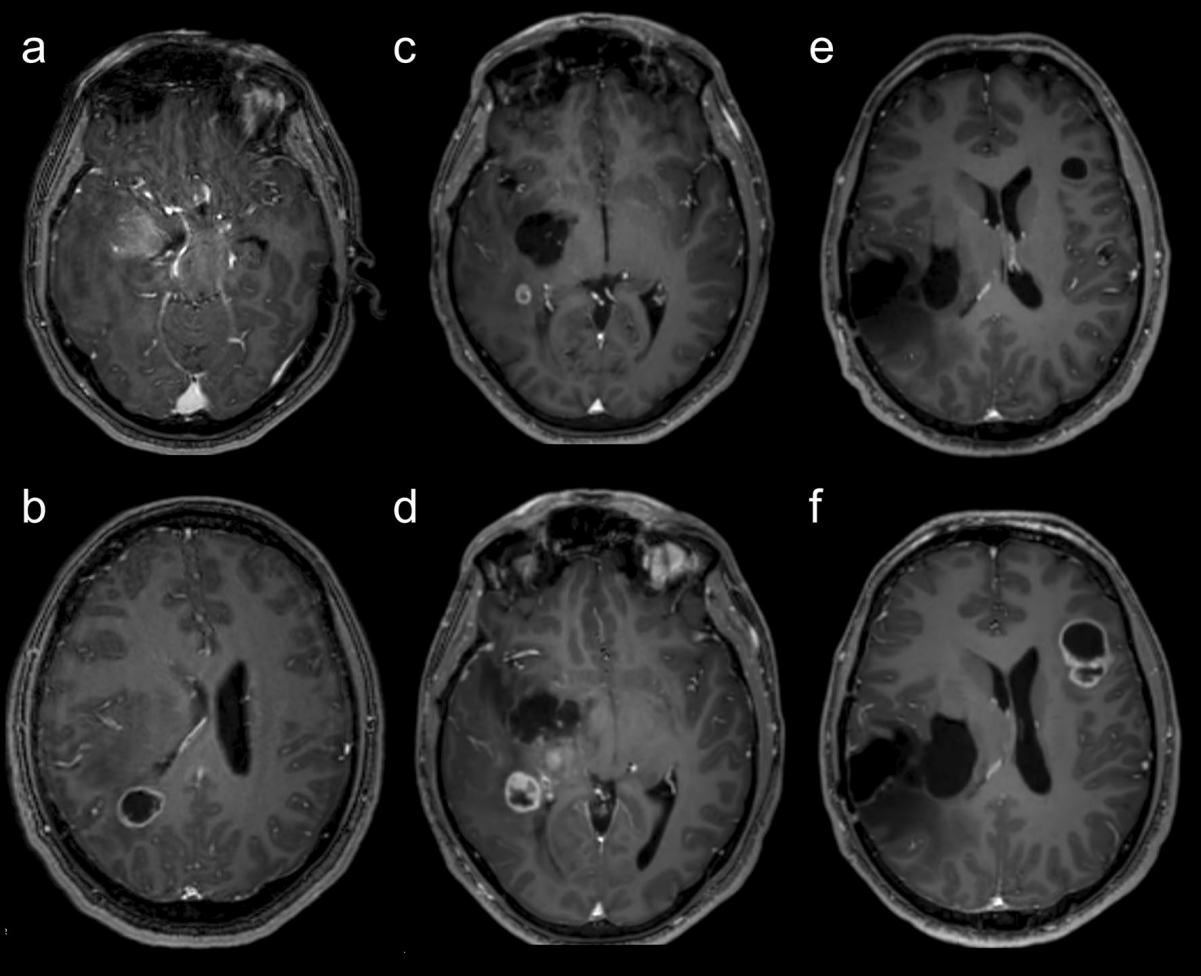
Radiological findings over the course of the patients’ treatment. Axial postcontrast T1 weighted magnetic resonance imaging (MRI) showing a right-sided inhomogeneous contrast enhancing lesion located in the basal ganglia and peritrigonal area (**a**), as well as a parietal cystoid mass (**b**). After initial partial resection right peritrigonal tumor progression was seen (**c** and **d**). Furthermore, another left frontal rapidly progressive cystic tumor developed (**e** and **f**)

A 42 year-old male Caucasian patient was diagnosed with two intracranial lesions due to headache and nausea. The larger lesion, located in the right temporomesial lobe, showed signs of intratumoral hemorrhage as well as contrast enhancement with associated perifocal edema and midline shift (Fig. 1a). The other tumor was a cystoid mass located in the trigonal area (Fig. 1b). Upon decision in the interdisciplinary neuro-oncological tumorboard and receival of written informed consent, the patient underwent resection of the temporomesial tumor via a transtemporal approach. Postoperative magnetic resonance imaging (MRI) revealed a subtotal resection with minimal residual ventral contrast-enhancement (Fig. 1c). Histopathological evaluation revealed an oligodendroglioma, *IDH1* mutated, 1p/19q-co-deleted WHO II, and a concomitant and adjuvant radiochemotherapy (50 Gy) with temozolomide (6 cycles) was initiated [11]. Initial follow-up imaging showed a stable temporomesial tumor and a decreased trigonal lesion. However, fifteen months after initial diagnosis a right-sided peritrigonal tumor progression was seen on MRI, and confirmed by [18F]fluoroethyltyrosine (FET)-PET CT (Fig. 1d). Due to only little mass effects of the progression, a wait-and-scan procedure was performed. However, in the subsequent MRIs, a new irregular circularly contrast enhancing lesion in the left frontal lobe was detected (Fig. 1e) sowing rapid tumor pregression (Fig. 1f). Re-resection of the right-sided tumor as well as the contralateral lesion was performed. The right frontotemporal lesion was now graded as an anaplastic oligodendroglioma, *IDH* mutated, 1p/19q co-deleted WHO III; and the left frontal tumor was classified as a glioblastoma *IDH* wildtype WHO IV. Subsequently the patient underwent re-irradiation with adjuvant bevacizumab therapy.

## Pathological findings

**Fig. 2:**
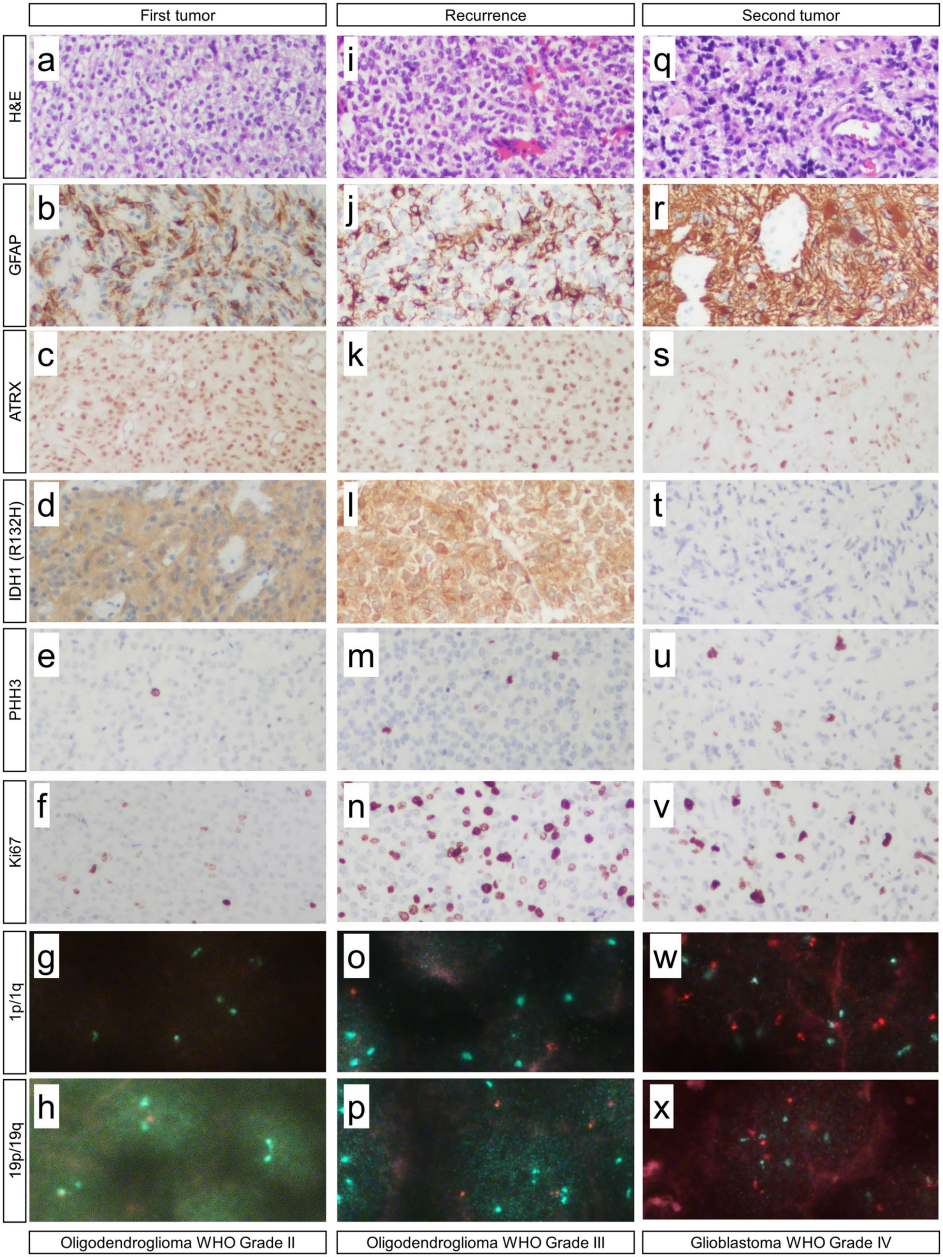
Histological and immunohistochemical findings. In H&E staining, the first tumor showed round shaped glial tumor cells with perinuclear halos (**a**). Immunohistochemistry with antibodies against GFAP showed positive tumor cells with only short processes (**b**). Reactions with antibodies against ATRX showed retained expression (**c**). Antibodies against IDH1 R132H mutant protein showed positive tumor cells (**d**). There were only sparse PHH3 positive cells (**e**). Proliferation was increased with 5% Ki67 positive cells (**f**). FISH analysis showed a combined loss of 1p (**g**) and 19q (**h**). Recurrence showed in H&E staining round tumor cells with perinuclear halos and brisk mitotic activity (**i**). Immunohistochemistry showed GFAP positive tumor cells (**j**). Nuclear expression of ATRX was retained (**k**). There was expression of IDH1 R132H mutant protein (**l**). There were increased PHH3 positive cells (**m**). Proliferation was increased with 25% Ki67 positive cells (**n**). FISH analysis of the 1p and 19q status revealed a combined loss of 1p (**o**) and 19q (**p**). Analysis of the second tumor showed in H&E staining a highly pleomorphic glia tumor with microvascular proliferation (**q**). Immunohistochemistry showed GFAP positive tumor cells (**r**). Nuclear expression of ATRX was retained (**s**). There was no expression of IDH1 R132H mutant protein (**t**). Reactions with antibodies against PHH3 showed increased mitoses (**u**). Proliferation was increased with 20% Ki67 positive cells (**v**). FISH analysis of the 1p and 19q status revealed no combined loss of 1p (**w**) and 19q (**x**)

The first manifestation showed in H&E staining a pleomorphic glial tumor with round tumor cells and perinuclear halos and only sparse mitoses (Fig. 2a). Immunohistochemistry performed on a Ventana Benchmark Ultra System with standard protocols showed that glial tumor cells were positive for GFAP (glial fibrillary acidic protein) with only short processes (Fig. 2b). Nuclear expression of ATRX (nuclear immunopositivity for α-thalassemia/mental-retardation-syndrome-X-linked) was retained (Fig. 2c), and there was expression of IDH1 (isocitrate dehydrogenase 1) R132H mutant protein (Fig. 2d). There were only sparse PHH3 (phosphorylated histone H3, H3S10p) positive cells (Fig. 2e) and proliferation was increased with 5% Ki67 positive cells (Fig. 2f). Analysis of the 1p and 19q status was performed by fluorescence in situ hybridization (FISH) using standard protocols, revealing a combined loss of 1p (Fig. 2g) and 19q (Fig. 2h). Thus, the tumor was classified as oligodendroglioma, *IDH* mutated, 1p/19q co-deleted, WHO grade II.

Recurrence of the right temporomesial lesion showed a similar picture as the first manifestation in H&E staining with round tumor cells and perinuclear halos but there was increased pleomorphy and brisk mitotic activity (Fig. 2i). Immunohistochemistry showed GFAP positive tumor cells with only short processes (Fig. 2j). Nuclear expression of ATRX was retained (Fig. 2k) and there was expression of IDH1 R132H mutant protein (Fig. 2l). There were increased PHH3 positive cells (Fig. 2m) and proliferation was increased with 25% Ki67 positive cells (Fig. 2n). Analysis of the 1p and 19q status performed by FISH revealed a combined loss of 1p (Fig. 2o) and 19q (Fig. 2p). Thus, this tumor was classified as recurrence of the previously described oligidendroglioma, then with features of anaplastic oligodendroglioma *IDH* mutated 1p/19q co-deleted WHO grade III.

Analysis of the left frontal lesion showed in H&E staining a highly pleomorphic glial tumor with long tumor processes, high mitotic activity and microvascular proliferation (Fig. 2q). Immunohistochemistry showed GFAP positive tumor cells with long processes (Fig. 2r). Nuclear expression of ATRX was retained (Fig. 2s). There was no expression of IDH1 R132H mutant protein (Fig. 2t). Reactions with antibodies against PHH3 showed increased mitoses (Fig. 2u). Proliferation was increased with 20% Ki67 positive cells (Fig. 2v). Analysis of the 1p and 19q status performed by FISH revealed no combined loss of 1p (Fig. 2w) and 19q (Fig. 2x). Thus, this tumor showed all the key hallmarks of a glioblastoma *IDH* wildtype WHO grade IV.

## Molecular genetic profiling

Molecular genetic analysis was performed by extracting DNA from FFPE material using the Maxwell system (Promega) according to the manufacturer’s protocol and subsequent application of the Illumina Focus Panel (Illumina) on an Illumina MiniSeq device (Illumina) according to the manufacturer’s protocols enabling us to analyze 41 genes in parallel, including *IDH1* and *IDH2* hot spot regions (the complete gene list can be found in Table 1). Hot spot loci of TERT promoter were analyzed by Sanger sequencing [4, 7]. DNA-methylation profiling was performed using Illumina EPIC bead chips that were scanned on an Illumina NextSeq 550DX device. Data analysis was performed using the Molecular Neuropathology Pipeline of the German Cancer Research Center (DKFZ) [1].

**Table 1:** AmpliSeq for ilumina focus panel gene list

AKT1	EGFR	GNA11	KRAS	PIK3CA
ALK	ERBB2	GNAQ	MAP2K1	RAF1
AR	ERBB3	HRAS	MAP2K2	RET
BRAF	ERBB4	IDH1	MET	ROS1
CCND1	ESR1	IDH2	MTOR	SMO
CDK4	FGFR1	JAK1	MYC	
CDK6	FGFR2	JAK2	MYCN	
CTNNB1	FGFR3	JAK3	NRAS	
DDR2	FGFR4	KIT	PDGFRA	

An overview of all 41 genes coveres using the AmpliSeq for illumina focus panel

**Fig. 3:**
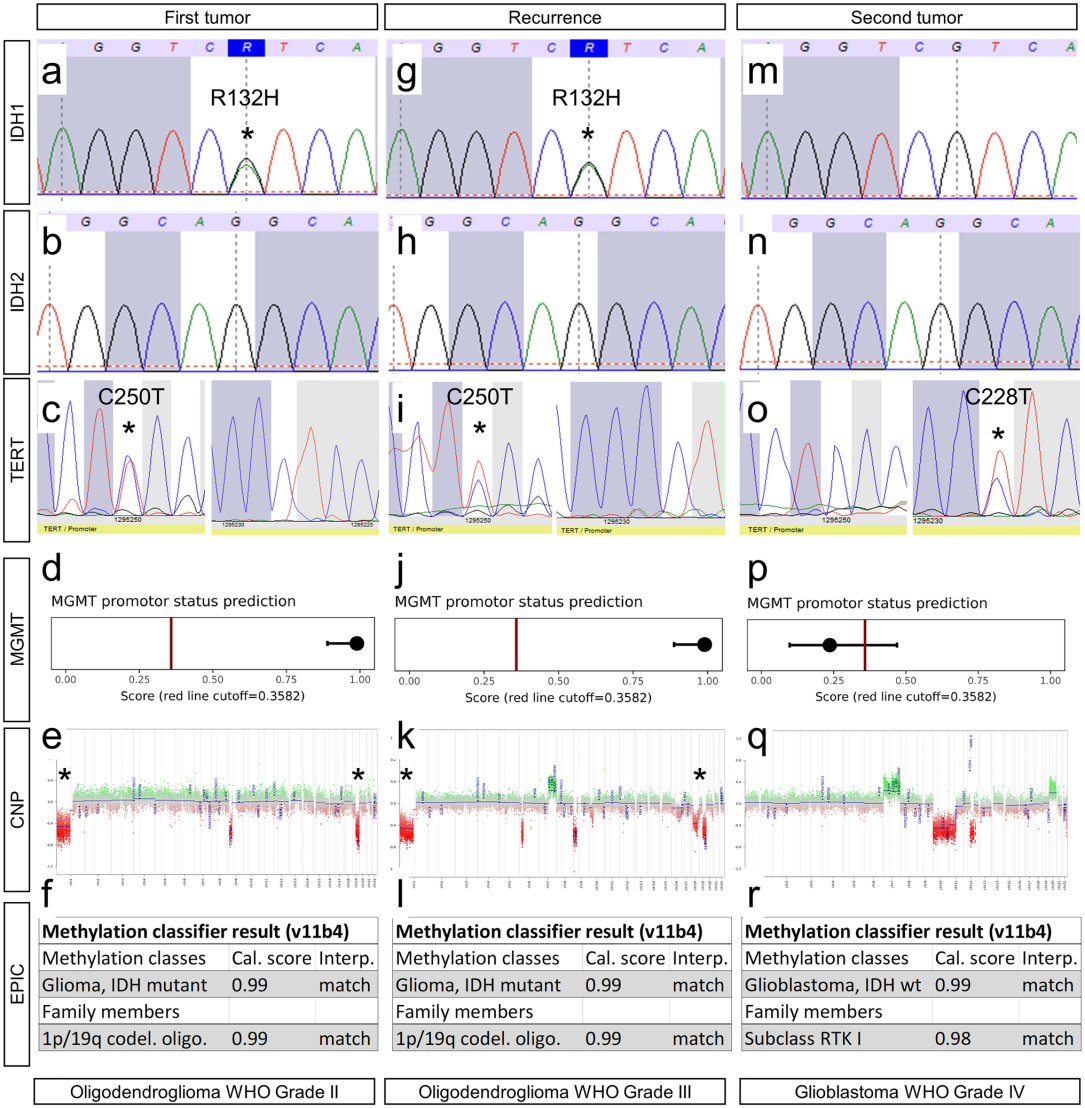
Molecular genetic findings. The first tumor manifestation showed an *IDH1* R132H mutation (**a**) with *IDH2* wildtype (**b**) and *TERT* C250T promoter mutation (**c**). DNA Methylation profiling showed methylated MGMT promoter (**d**), 1p and 19q losses in CNP (**e**) and allocated the tumor to the methylation class of oligodendroglioma IDH mutated 1p/19q co-deleted (**f**). The recurrence showed *IDH1* R132H mutation (**g**) with *IDH2* wildtype (**h**) and *TERT* C250T promoter mutation (**i**). DNA-methylation profiling showed methylated MGMT promoter (**j**), 1p and 19q losses in CNP (**k**) and allocated the tumor to the methylation class of oligodendroglioma IDH mutated 1p/19q co-deleted (**l**). The second tumor showed *IDH1* wildtype (**m**), *IDH2* wildtype (**n**) and *TERT* C228T promoter mutation (**o**). DNA Methylation profiling showed unmethylated MGMT promoter (**p**), no 1p and 19q loss in CNP (**q**) and allocated the tumor to the methylation class of glioblastoma IDH wildtype, subclass RTK I (r). *: indication of *IDH1* and *TERT* mutations and 1p/19q losses

Integrated work-up of the first tumor manifestation showed an *IDH1* R132H mutation (Fig. 3a) with *IDH2* wildtype (Fig. 3b) and *TERT* C250T promoter mutation (Fig. 3c). DNA Methylation profiling showed methylated MGMT promoter (Fig. 3d), 1p and 19q losses in copy number profiling (Fig. 3e) and allocated the tumor to the methylation class of oligodendroglioma IDH mutated 1p/19q co-deleted (Fig. 3f).

Analysis of the recurrence revealed an analogous molecular profile: The tumor showed an *IDH1* R132H mutation (Fig. 3g) with *IDH2* wildtype (Fig. 3h) and *TERT* C250T promoter mutation (Fig. 3i). DNA Methylation profiling showed methylated MGMT promoter (Fig. 3j), 1p and 19q losses in copy number profiling (Fig. 3k) and allocated the tumor to the methylation class of oligodendroglioma *IDH* mutated 1p/19q co-deleted (Fig. 3l).

Interestingly, profiling of the left-sided tumor manifestation revealed a fundamentally different profile: This tumor showed *IDH1* (Fig. 3m) and *IDH2* wildtype (Fig. 3n) and *TERT* C228T promoter mutation (Fig. 3o).

DNA Methylation profiling showed unmethylated MGMT promoter (Fig. 3p); there was no 1p and 19q loss in copy number profiling (Fig. 3q) and allocated the tumor to the methylation class of glioblastoma *IDH* wildtype, subclass RTK I (Fig. 3r).

All other 40 genes covered by the AmpliSeq for Illumina Gene Panel showed an identical gene alteration profile in all three tumors (Table 2).

**Table 2:** Detected gen alterations

First tumor				Recurrence				Second tumor				Prediction
Gene	Allele frequency (reads) [forward/reverse]	c. HGVS	p. HGVS	Gene	Allele frequency (reads) [forward/reverse]	c. HGVS	p. HGVS	Gene	Allele frequency (reads) [forward/reverse]	c. HGVS	p. HGVS	Mutational effect
*ALK*	100% (8978) [100% (4363)/100% (4615)]	c.4381A > G	p.Ile1461Val	*ALK*	100% (8530) [100% (4109)/100% (4421)]	c.4381A > G	p.Ile1461Val	*ALK*	100% (6466) [100% (3128)/100% (3338)]	c.4381A > G	p.Ile1461Val	Class 1 (benign)
*DDR2*	31% (1079) [31% (542)/31% (537)]	c.278C > T	p.Thr93Ile	*DDR2*	26% (862) [26% (430)/27% (432)]	c.278C > T	p.Thr93Ile	*DDR2*	19% (579) [19% (294)/19% (285)]	c.278C > T	p.Thr93Ile	
*EGFR*	29% (1418) [31% (627)/28% (791)]	c.89-10986delT		*EGFR*	26% (1358) [29% (591)/24% (767)]	c.89-10986delT		*EGFR*	26% (1579) [29% (696)/25% (883)]	c.89-10986delT		
*EGFR*	52% (2986) [52% (1465)/51% (1521)]	c.1498 + 22A > T		*EGFR*	53% (2637) [54% (1297)/52% (1340)]	c.1498 + 22A > T		*EGFR*	34% (1794) [35% (884)/33% (910)]	c.1498 + 22A > T		Class 1 (benign)
*ERBB3*	100% (4558) [100% (2337)/100% (2221)]	c.234 + 8A > T		*ERBB3*	100% (5482) [100% (2817)/100% (2665)]	c.234 + 8A > T		*ERBB3*	100% (5267) [100% (2687)/100% (2580)]	c.234 + 8A > T		
*FGFR3*	100% (4370) [100% (2228)/100% (2142)]	c.1953G > A	p.Thr651=	*FGFR3*	100% (3469) [100% (1794)/100% (1675)]	c.1956G > A	p.Thr652=	*FGFR3*	99% (2811) [100% (1457)/99% (1354)]	c.1956G > A	p.Thr652=	Class 1 (benign)
*FGFR4*	48% (2725) [49% (1420)/47% (1305)]	c.92-65 T > C		*FGFR4*	51% (3086) [52% (1577)/51% (1509)]	c.92-65 T > C		*FGFR4*	53% (2405) [54% (1231)/53% (1174)]	c.92-65 T > C		
*FGFR4*	49% (1362) [49% (697)/48% (665)]	c.407C > T	p.Pro136Leu	*FGFR4*	50% (1956) [50% (987)/50% (969)]	c.407C > T	p.Pro136Leu	*FGFR4*	48% (1747) [48% (895)/48% (852)]	c.407C > T	p.Pro136Leu	Class 1 (benign)
*FGFR4*	54% (833) [54% (432)/53% (401)]	c.483A > G	p.Ala161=	*FGFR4*	54% (1041) [54% (537)/54% (504)]	c.483A > G	p.Ala16=	*FGFR4*	55% (1115) [55% (573)/56% (542)]	c.483A > G	p.Ala161=	
*FGFR4*	46% (265) [48% (141)/45% (124)]	c.2016-43C > A		*FGFR4*	45% (365) [47% (190)/44% (175)]	c.1896-43C > A		*FGFR4*	50% (462) [52% (237)/49% (225)]	c.1896-43C > A		
*FGFR4*	48% (272) [50% (137)/47% (135)]	c.2016-8A > G		*FGFR4*	48% (374) [52% (185)/45% (189)]	c.1896-8A > G		*FGFR4*	53% (475) [56% (233)/50% (242)]	c.1896-8A > G		
*IDH1*	46% (5687) [46% (2808)/46% (2879)]	c.395G > A	p.Arg132His	*IDH1*	48% (5691) [48% (2838)/48% (2853)]	c.395G > A	p.Arg132His	*IDH1*	–	–	–	Class 5 (pathogenic)
*KIT*	48% (4257) [47% (2140)/49% (2117)]	c.67 + 4913A > G		*KIT*	48% (3192) [47% (1615)/48% (1577)]	c.67 + 4913A > G		*KIT*	46% (2393) [45% (1213)/46% (1180)]	c.67 + 4913A > G		
*KIT*	23% (2198) [23% (1076)/23% (1122)]	c.67 + 4923delA		*KIT*	21% (1505) [21% (742)/21% (763)]	c.67 + 4923delA		*KIT*	22% (1249) [23% (615)/22% (634)]	c.67 + 4923delA		
*KIT*	43% (4173) [37% (1745)/49% (2428)]	c.67 + 4953dupA		*KIT*	43% (3102) [37% (1306)/49% (1796)]	c.67 + 4953dupA		*KIT*	42% (2340) [36% (995)/47% (1345)]	c.67 + 4953dupA		
*KIT*	100% (9294) [100% (4544)/100% (4750)]	c.756 + 334G > A		*KIT*	100% (7169) [100% (3547)/100% (3622)]	c.756 + 334G > A		*KIT*	100% (5445) [100% (2678)/100% (2767)]	c.756 + 334G > A		
*KIT*	50% (4812) [50% (2414)/50% (2398)]	c.2362-333A > T		*KIT*	50% (4707) [50% (2358)/50% (2349)]	c.2362-333A > T		*KIT*	50% (4102) [50% (2048)/50% (2054)]	c.2362-333A > T		
*KRAS*	51% (1012) [51% (496)/52% (516)]	c.-11-1877C > A		*KRAS*	44% (859) [43% (418)/44% (441)]	c.-11-1877C > A		*KRAS*	50% (1081) [50% (539)/50% (542)]	c.-11-1877C > A		
*KRAS*	50% (6037) [50% (3022)/51% (3015)]	c.111 + 6969C > G		*KRAS*	46% (4123) [46% (2061)/46% (2062)]	c.112-3079C > G		*KRAS*	49% (3521) [49% (1762)/49% (1759)]	c.112-3079C > G		
*PDGFRA*	100% (6940) [100% (3405)/100% (3535)]	c.1701A > G	p.Pro567=	*PDGFRA*	100% (7371) [100% (3629)/100% (3742)]	c.1701A > G	p.Pro567=	*PDGFRA*	100% (6090) [100% (3020)/100% (3070)]	c.1701A > G	p.Pro567=	Class 1 (benign)
*PIK3CA*	12% (2249) [12% (1141)/12% (1108)]	c.2119G > A	p.Glu707Lys	*PIK3CA*	12% (1979) [12% (1004)/12% (975)]	c.2119G > A	p.Glu707Lys	*PIK3CA*	12% (1470) [12% (751)/11% (719)]	c.2119G > A	p.Glu707Lys	Class 3 (uv)
*PIK3CA*	23% (4396) [23% (2182)/23% (2214)]	c.2155C > G	p.Leu719Val	*PIK3CA*	22% (3717) [22% (1846)/23% (1871)]	c.2155C > G	p.Leu719Val	*PIK3CA*	22% (2822) [22% (1395)/23% (1427)]	c.2155C > G	p.Leu719Val	Class 2 (likely benign)
*PIK3CA*	24% (4435) [24% (2215)/23% (2220)]	c.2187 + 1G > T		*PIK3CA*	23% (3688) [22% (1804)/23% (1884)]	c.2187 + 1G > T		*PIK3CA*	23% (2813) [23% (1380)/23% (1433)]	c.2187 + 1G > T		Class 3 (uv)
*RET*	50% (3715) [51% (1930)/48% (1785)]	c.2307G > T	p.Leu769=	*RET*	50% (3281) [51% (1692)/49% (1589)]	c.2307G > T	p.Leu769=	*RET*	13% (406) [13% (209)/13% (197)]	c.2307G > T	p.Leu769=	Class 1 (benign)

An overview of all detected gene alterations using the AmpliSeq for Illumina Focus Panel. All three tumors showed the same gene alterations except for the IDH1 R132H mutation (indicated in red color) that was not detected in the second tumor. Note that TERT mutations are not indicated here since TERT hot spot loci were analyzed by Sanger sequencing

## Discussion

Here we report on an unique case of a patient that developed two molecularly independent gliomas: oligodendroglioma and glioblastoma.

To our knowledge, this is the first reported case of a patient with two independent gliomas of oligodendroglioma and glioblastoma biology that were confirmed by integrated in-depth molecular profiling including epigenomic DNA-methylation analysis.

A literature search revealed only one other published case of a cerebellar glioblastoma and a supratentorial oligodendroglioma [6]. Junaid et al. reported on a 44-years old patient, who suffered from a cerebellar glioma with typical histological features of glioblastoma, i.e. microvascular proliferation and necrosis, and a supratentorial glioma with histological hallmarks of an oligodendroglioma, i.e. small round cells with perinuclear halos [6]. However, only a conventional histological work-up of the specimens had been performed, and no immunohistochemical and molecular profiling to prove different biological background of the two reported gliomas had been provided [6].

In the case presented here, it is astonishing, that the completely removed WHO Grade II oligodendroglioma recurred after only 15 weeks after radiochemotherapy. This might be due to hypermutations occurred by temozolomide chemotherapy. Of the 41 genes covered by the AmpliSeq for Illumina Focus Panel (Table 1), we did not find any changes in the gene alteration profile in the first tumor and the recurrence (Table 2), however, this panel may be too small to answer the question of hypermutations occurring after temozolomide chemotherapy and a larger gene panel may be appropriate. Furthermore, the question rises if there were any germline mutations in the patients. Germline mutations may be an important co-factor in this unique case showing recurrence and progression of a WHO Grade II oligodendroglioma after only 15 weeks and a molecularly independent WHO Grade IV glioblastoma. Unfortunately, we did not have the chance to check for germline mutations in the presented case.

In summary, our presented case is an unique example of a patient with two different gliomas proved by in-depth molecular work-up. Besides different histology of oligodendroglioma and glioblastoma, the two brain tumors showed different molecular profiles of oligodendroglioma (i.e. *IDH1* R132H mutation, combined 1p/19q loss, *TERT* C250T mutation) and glioblastoma (i.e. *IDH1* wildtype, retained 1p/19q, *TERT* C228T mutation), respectively. Additionally, epigenomic DNA-methylation profiling clustered the tumors to the classes of oligodendroglioma IDH mutant 1p/19q co-deleted and glioblastoma IDH wildtype subclass RTK I.

Thus, this unique case emphasizes the need for integrated molecular work-up and demonstrates the power of in-depth profiling including DNA-methylation profiling in better understanding tumor biology and revealing tumor heterogeneity.
